# P02.31. Effective and viable mind-body stress reduction in the workplace: two RCTs

**DOI:** 10.1186/1472-6882-12-S1-P87

**Published:** 2012-06-12

**Authors:** R Wolever, K Bobinet, K McCabe, L MacKenzie, E Fekete, C Kusnick, M Baime

**Affiliations:** 1 Duke University, Durham, USA; 2 Aetna, Walnut Creek, USA; 3 eMindful, Vero Beach, USA; 4 Univ of Pennsylvania, Philadelphia, USA; 5 University of Indianapolis, Indianapolis, USA; 6 Headlands Consulting, Orange County, USA; 7 University of Pennsylvania School of Medicine, Philadelphia, USA

## Purpose

Highly stressed employees are subject to greater health risks, costs, and productivity losses than those with normal stress levels. To address this issue, work-site stress management programs must be able to engage individuals as well as capture data on stress, health indices, work productivity, and healthcare costs. In this randomized controlled pilot, our primary objective was to evaluate the viability and proof of concept for two innovative mind-body workplace stress reduction programs, setting the stage for larger cost-effectiveness trials. A second objective was to evaluate two delivery venues of the mindfulness intervention (online versus in-person).

## Methods

Two-hundred thirty-nine (239) employee volunteers were randomized into a therapeutic Viniyoga worksite stress reduction program, one of two Mindfulness at Work™ programs, or a control group that participated only in assessment. Intention-to-treat principles and 2 (pre and post) X 2 (group) Repeated Measures ANCOVA procedures examined group differences over time on perceived stress and secondary measures to clarify which variables to include in future studies: sleep quality, mood, pain levels, work productivity, mindfulness, blood pressure, breathing rate, and heart rate variability (a measure of autonomic balance).

## Results

Compared to the control group, the mind-body interventions both showed significantly greater improvements on perceived stress, sleep quality and the heart rhythm coherence ratio of heart rate variability. The yoga program also demonstrated improved DBP and pain ratings. The mindfulness program improved mindfulness measures. The two delivery venues for the mindfulness program were basically equivalent in terms of outcomes, though study retention was better for the online venue.

## Conclusion

Both the Mindfulness at Work™ and therapeutic Viniyoga programs provide viable and effective interventions to target high stress levels, sleep quality and autonomic balance in employees. Future studies need to evaluate the impact on employer costs.

